# The Anti-Tumor Histone Deacetylase Inhibitor SAHA and the Natural Flavonoid Curcumin Exhibit Synergistic Neuroprotection against Amyloid-Beta Toxicity

**DOI:** 10.1371/journal.pone.0085570

**Published:** 2014-01-07

**Authors:** Jia Meng, Yan Li, Cynthia Camarillo, Yue Yao, Yina Zhang, Chun Xu, Lihong Jiang

**Affiliations:** 1 Department of Geriatrics, the Second Affiliated Hospital of Harbin Medical University, Harbin, Heilongjiang, China; 2 Department of Pharmacy, the Fourth Affiliated Hospital of Harbin Medical University, Harbin, Heilongjiang, China; 3 The Center of Excellence in Neuroscience, Texas Tech University Health Sciences Center, El Paso, Texas, United States of America; Peking University Health Science Center, China

## Abstract

With the trend of an increasing aged population worldwide, Alzheimer's disease (AD), an age-related neurodegenerative disorder, as one of the major causes of dementia in elderly people is of growing concern. Despite the many hard efforts attempted during the past several decades in trying to elucidate the pathological mechanisms underlying AD and putting forward potential therapeutic strategies, there is still a lack of effective treatments for AD. The efficacy of many potential therapeutic drugs for AD is of main concern in clinical practice. For example, large bodies of evidence show that the anti-tumor histone deacetylase (HDAC) inhibitor, suberoylanilidehydroxamic acid (SAHA), may be of benefit for the treatment of AD; however, its extensive inhibition of HDACs makes it a poor therapeutic. Moreover, the natural flavonoid, curcumin, may also have a potential therapeutic benefit against AD; however, it is plagued by low bioavailability. Therefore, the integrative effects of SAHA and curcumin were investigated as a protection against amyloid-beta neurotoxicity in vitro. We hypothesized that at low doses their synergistic effect would improve therapeutic selectivity, based on experiments that showed that at low concentrations SAHA and curcumin could provide comprehensive protection against Aβ_25–35_-induced neuronal damage in PC12 cells, strongly implying potent synergism. Furthermore, network analysis suggested that the possible mechanism underlying their synergistic action might be derived from restoration of the damaged functional link between Akt and the CBP/p300 pathway, which plays a crucial role in the pathological development of AD. Thus, our findings provided a feasible avenue for the application of a synergistic drug combination, SAHA and curcumin, in the treatment of AD.

## Introduction

Alzheimer's disease (AD) is the main cause of dementia [1]. It is pathologically defined as an age-related progressive neurodegenerative disorder and characterized by the extracellular accumulation of amyloid-beta (Aβ) plaques [2], the intracellular aggregation of neurofibrillary tangles (NFTs) formed by hyperphosphorylated tau protein [3]. Although there are many medicines available for the treatment of AD in the clinic, such as the acetylcholinesterase inhibitor, rivastigmine, and the low-affinity NMDA (N-methyl-d-aspartate) receptor antagonist, memantine, neither one of these can really treat the underlying causes of AD except for just rendering some modest symptomatic improvement. With the trend of an increasing aged population worldwide and due to the lack of effective treatments, it is pessimistically estimated that AD might overwhelm the society and health-care systems in the near future [4].

Current amyloid-β-directed therapeutics were thought of as the most promising strategy for AD; however, they have been failing Phase III clinical trials, this leads to doubt amyloid-β as the leading player in the amyloid cascade hypothesis, which has profoundly affected the AD field for two decades [5], [6]. Some scholars suggest that the current amyloid cascade hypothesis is still correct but that some modification is needed [4]. Amyloid-β is considered as a trigger for the pathogenesis of AD but it is not the major disease driver. Taken together, amyloid-β-directed therapeutics fail to undermine the complexity of AD pathology which suggests that alternative therapeutic strategies should be proposed with the premise of an in-depth understanding of AD pathology and treatment response.

Recent gene expression and functional studies have demonstrated that significant chromosomal alterations of histone acetylation and DNA methylation might play a vital role in initiating the pathogenesis of AD [7]–[9]. In human cells, histone acetyltransferases (HATs) and histone deacetylases (HDACs) catalyze histone acetylation and deacetylation, respectively; thus, the vital role in adjusting histone acetylation to healthy levels, and targeting AD-associated abnormal histone acetylation make HDACs rational therapeutic targets [10]. Actually, it has been experimentally validated that the HDAC inhibitor, suberoylanilidehydroxamic acid (SAHA), truly improved memory and cognition in an AD animal model, suggesting it as a promising treatment for AD [11]. However, SAHA is classified as a non-selective HDAC inhibitor due to its extensive targeting of HDAC proteins including HDAC1, HDAC2, HDAC3, HDAC6, and HDAC8 [12]. This leads to widespread worries by research scholars about the therapeutic selectivity of SAHA as a treatment for AD [13], [14].

Based upon the confirmatory conclusion that the therapeutic selectivity of one drug could be significantly improved when in a synergistic drug combination [15], we proposed here that SAHA, as an alternative treatment for AD, is feasible, but only if a clinically available drug could be found to act synergistically with it. With this working hypothesis, we examined whether SAHA and the natural flavonoid, curcumin, could synergistically protect against Aβ-induced neuronal apoptosis in PC12 cells. The latter has been shown to protect against neuronal injuries [16]. In the present study, we sought to validate the synergistic neuroprotection effect of SAHA and curcumin. Our in vitro experiments indicated a potent synergistic neuroprotection effect exists when SAHA is in combination with curcumin at low concentration levels. Furthermore, we also attempted to clarify the synergistic mechanism by integrating public resources for performing network analysis of global gene expression in AD [7]. Specifically, our results collectively indicated that the CBP/p300 signaling pathway might play a dominant role in the progression of AD. However, further dissection of protein connectivity suggested that the activity of Akt was necessarily required for functional execution of CBP/p300 in tuning gene transcription. We further confirmed that the interaction between Akt and the CBP/p300 signaling pathway was vital to the repair of Aβ-induced neuronal injury during co-treatment of SAHA and curcumin.

In the present study, we validated the synergistic neuroprotection of SAHA and curcumin. The possible mechanism underlying the synergistic action was also revealed. In addition, since both SAHA and curcumin are commercially available, this makes our proposed synergistic drug therapeutic strategy for AD treatment accessible. Overall, our finding provides a rationale avenue for clinical implementation of SAHA in selectively treating AD in the current clinical context.

## Materials and Methods

### Cell Culture and Treatment

Rat pheochromocytoma PC12 cells were originally obtained from Chinapeptides Co., Ltd. PC12 neuron cells were cultured in DMEM-F12 containing 7% FBS, 1% penicillin, and 1% streptomycin at 37°C. Before the drug treatment, cells were cultured in serum-free medium for 12 h. SAHA (1 µM) and curcumin (5 µM) were then added alone or together 1 h prior to adding the Aβ25–35 (20 µM) treatment. The cells were then incubated for another 24 h in a humidified incubator in the presence of 95% O_2_ and 5% CO_2_.

### MTT Assay

MTT assay was used to measure the viability of PC12 cells. Briefly, PC12 cells were plated in a 96-well plate in DMEM-F12 with 7% FBS. After culturing in serum-free DMEM-F12 for 12 h, Aβ25–35 (20 µM) alone or in combination with SAHA,curcumin,or SAHA+curcumin were added to the medium. Twenty-four hours later, PC12 cells were incubated with 10 µL MTT (Roche, 0.5 mg/ml) at 37° for 4 h, and then the crystals were dissolved with 150 µL DMSO. Absorbance at 570 nm was detected by a spectrophotometer (TECAN Infinite M200, Switzerland).

### Acridine Orange/ethidium Bromide (AO/EB) Staining

To detect the impact of SAHA (1 µM) and curcumin (5 µM) on Aβ25–35 (20 µM) mediated cell apoptosis, the cultured PC12 cells were washed with PBS and then incubated with 100 µg/mL of AO and EB (Sigma Aldrich, USA) for 5 min. Normal and apoptotic cells were observed under a fluorescence microscope equipped with a CCD digital camera (Nikon Corporation, Japan).

### Superoxide Dismutase Activity (SOD) Measurement

The activity of SOD in PC12 cells was measured using the SOD Detection kit (Nanjing Jiancheng Bioengineering Institute, China). Following the manufacturer’s instruction, the results were determined at 550 nm and are expressed as unit per mgprot.

### TdT-mediated dUTP Nick End Labeling (TUNEL) Staining

For apoptosis analysis, DNA fragmentation of PC12 cells was measured with an in situ Cell Death Detection Kit (Roche Diagnostics Corporation, Indianapolis, IN, USA), according to the manufacturer’s instructions. Followed by TUNEL staining, PC12 cell nuclei were counterstained with DAPI (Sigma-Aldrich, St Louis, MO, USA). With a fluorescence microscope (Nikon 80i, Japan), TUNEL positive PC12 cells were counted in 5 randomly selected fields (200× magnification), respectively, and the average rate of apoptotic cells was calculated.

### Western Blot Analysis

Protein samples extracted from the PC12 cells were used for immunoblotting analysis. Briefly, samples were fractionated by SDS-PAGE (10% polyacrylamide gels), transferred to nitrocellulose membrane (Millipore, Bedford, MA, USA) and incubated with primary antibodies against Akt (Cell Signaling Technology, Inc. USA), p(Ser 473)-Akt (Cell Signaling Technology, Inc. USA), cleaved caspase 3 (Cell Signaling Technology, Inc. USA) and β-actin (KangchengInc, China) at 4°C overnight. The membranes were washed and incubated with secondary antibodies for 1 hour at room temperature. Finally, the bands were quantified with the Odyssey v1.2 software by measuring intensity (area×OD); β-actin was used as the internal control.

### Gene Expression Correlation Analysis

The gene microarray dataset (accession number: GSE1297) was retrieved from the Gene Expression Omnibus (GEO) database [7]. The 22 disease hippocampus samples were divided into three groups according to the stages of AD. The gene expression value was only accepted if detected as 'present' or 'marginal' on at least 16 chips. The likelihood of the normal distribution of the values of MiniMental Status Examination (MMSE) and neurofibrillary tangle (NFT) scores was assessed using the D'Agostino & Pearson omnibus normality test. MMSE scores were of normal distribution (*p* = 0.183), so where the NFT scores after square root transformation (*p* = 0.795). Pearson linear correlation assessment was applied for the median-normalized expression values (log_2_) of each gene with MMSE score and the square root of NFT score, respectively at different stages of AD. The Benjamini and Hochberg method of multiple testing [17] was then applied to adjust the p-values for genes if values of the square of correlation coefficient R^2^ were more than 0.30 for the MMSE score or for the square root of NFT score at any disease stage. Significant AD relation was only accepted when the adjusted p-value was less than 0.05.

### Network Topology Calculation

By retrieving experimentally validated PPIs [18], Cytoscape [19] generated a human hippocampus-specific PPI network (hHPIN) for the proteins expressed in human hippocampus [20]. Single nodes and small network components were removed and so were the self-loop edges. The pruning was done in order to analyze the giant component of the network. The topological parameters, degree and betweenness centrality were calculated [21] to identify hubs (degree >50) and nodes that exerted extensive control over PPIs (betweenness centrality >5×10^−4^).

### Gene Ontology (GO) Analysis

The DAVID functional annotation clustering tool was applied to detect significantly over-represented biological processes for the up- and down-regulated AD-related genes, respectively (Enrichment Score >2.0) [22]. All tool options were set as default except when the threshold of Ease Score was 0.05 and the number of genes was more than ten. As the gradual change of disease-related processes reflected the evolution trajectory of the AD pathology to a certain extent, genes of abnormal expression were counted at different disease stages for each process.

### GenePro and Expression Distance Analyses

Different proteins can jointly participate in one process. Here, we hypothesize that two processes might be related functionally if experimental evidence suggested that protein A in a process interacted with protein B in another process. GenePro was applied to investigate the functional link between AD-related processes [23]. More PPIs might mean closer functional link between two processes. A geometry distance formula of 2 points in a three-space was applied to calculate the gene expression distance between different processes. Simply, to a given process the percentages of its process genes at early-, medium- and late-stage of AD were symbolized as the three-space point coordinate x, y and z, respectively. Short gene expression distances indicated a coordinated gene expression trend was adopted by two processes.

### Statistical Analysis

All data are expressed as means ± SEM. Statistical analysis was performed using one-way ANOVA followed by correction for multiple testing using Bonferroni’s method. Differences were considered as statistically significant when *p*<0.05.

## Results

### The Combination of SAHA and Curcumin Improved Cellular Viability of Aβ-treated PC12 Cells

**Figure 1:**
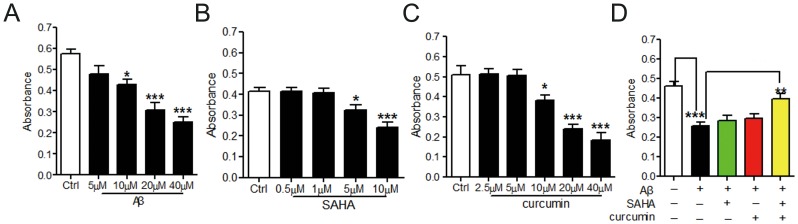
Effect of SAHA and curcumin on cell viability of PC12 cells. A. PC12 cells were treated with different concentrations of Aβ_25–35_ (n = 5). *, *p*<0.05; ***, *p*<0.001 vs Ctrl. B. PC12 cells were treated with different concentrations of SAHA (n = 5). *, *p*<0.05; ***, *p*<0.001 vs Ctrl. C. PC12 cells were treated with different concentrations of curcumin (n = 5). *, *p*<0.05; ***, *p*<0.001 vs Ctrl. D. MTT assay was performed to detect cell viability after treating with SAHA and curcumin against Aβ_25–35_-induced cytotoxicity in PC12 cells (n = 5). ***, *p*<0.001 vs Ctrl; **, *p*<0.01 vs Aβ_25–35_.

**Figure 2:**
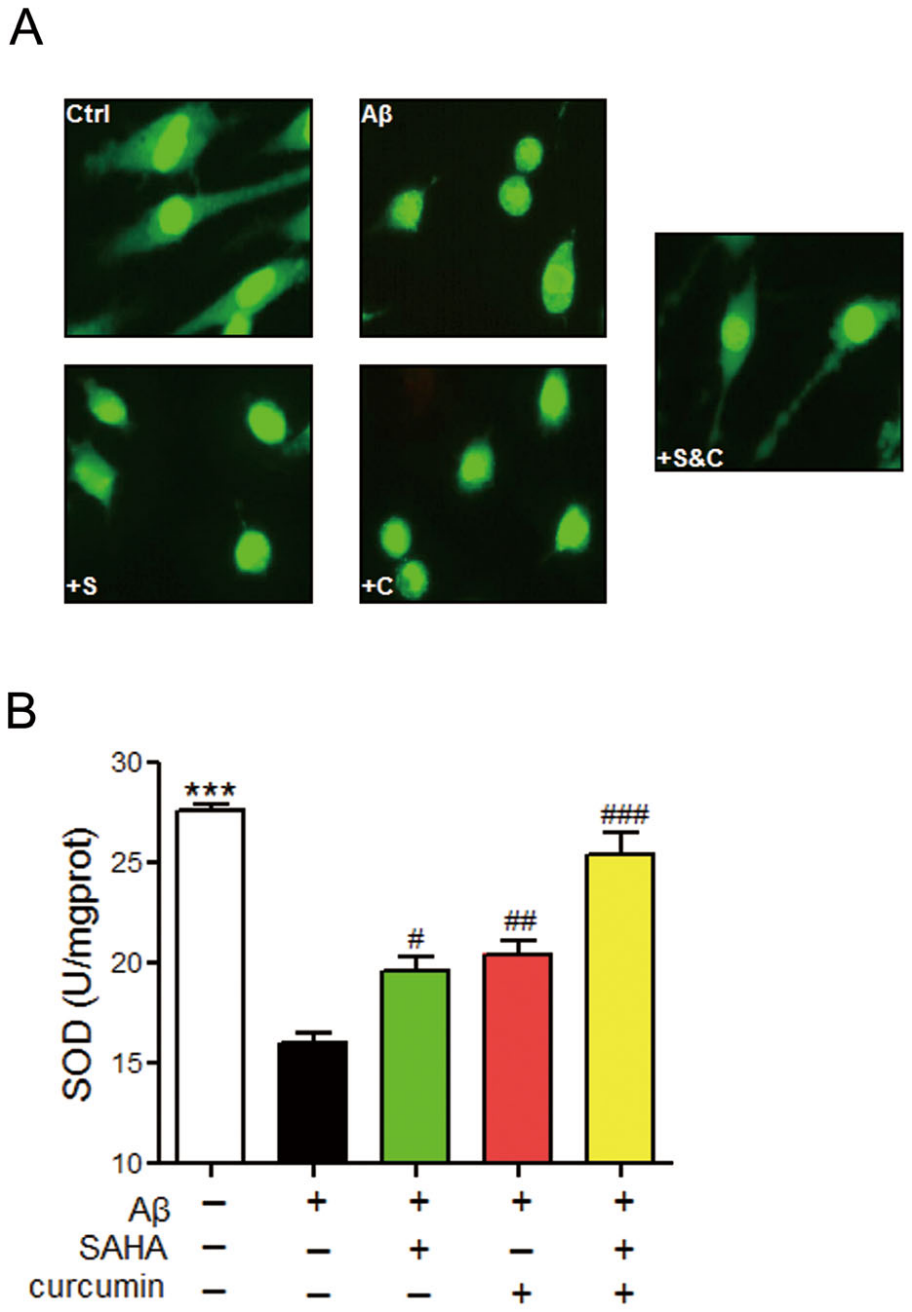
Effect of SAHA and curcumin on neuronal integrity and oxidative stress. A. Result of AO/EB staining. S: SAHA; C: curcumin. B. Result of SOD content measurement. ***, *p*<0.001 vs Ctrl; #, *p*<0.05; ##, *p*<0.01, ###, *p*<0.001, vs Aβ_25–35_.

Increasing concentrations of Aβ_25–35_, SAHA, and curcumin alone were able to induce obvious cytotoxicity in PC12 cells as indicated by the MMT assay (Figure 1A–C). The respective concentrations of SAHA and curcumin were optimally selected at 1 and 5 µM for the following evaluation of their potential synergistic effect against Aβ_25–35_-induced neuronal injury. Despite that SAHA at 1 µM and curcuminat 5 µM showed no apparent cytotoxicity in PC12 cells, we did not observe an obvious improvement in cell viability after using SAHA or curcumin treatment alone (Figure 1D). This result was further confirmed because the selected concentrations of SAHA and curcumin were too low to obtain any acceptable neuronal cell restoration from Aβ_25–35_-induced damage as AO/EB staining and SOD measurement indicated (Figure 2).

Interestingly, we found that a significant relief from Aβ_25–35_-induced neuronal injury could be clearly achieved by applying a combination of 1 µM SAHA and 5 µM curcumin (*p*<0.01) in PC12 cells, implying potential synergy (Figure 1D). In addition to improving neuronal viability, co-treatment of SAHA and curcumin could also successfully restore the structural integrity of the neuronal cells and intracellular oxidative stress environment that was caused by excessive deposition of Aβ (Figure 2). This finding definitely suggests that the monotherapy of the anti-tumor HDAC inhibitor SAHA is not a rational treatment avenue for reversing AD pathology due to poor therapeutic specificity at a low dose and significant neurotoxicity at a high dose. However, co-treatment of SAHA and curcumin could be a better choice as a result of the synergistic protection against Aβ-mediated decline in neuronal function.

### SAHA and Curcumin Synergistically Inhibit Aβ-induced Cellular Apoptosis in PC12 Cells

**Figure 3:**
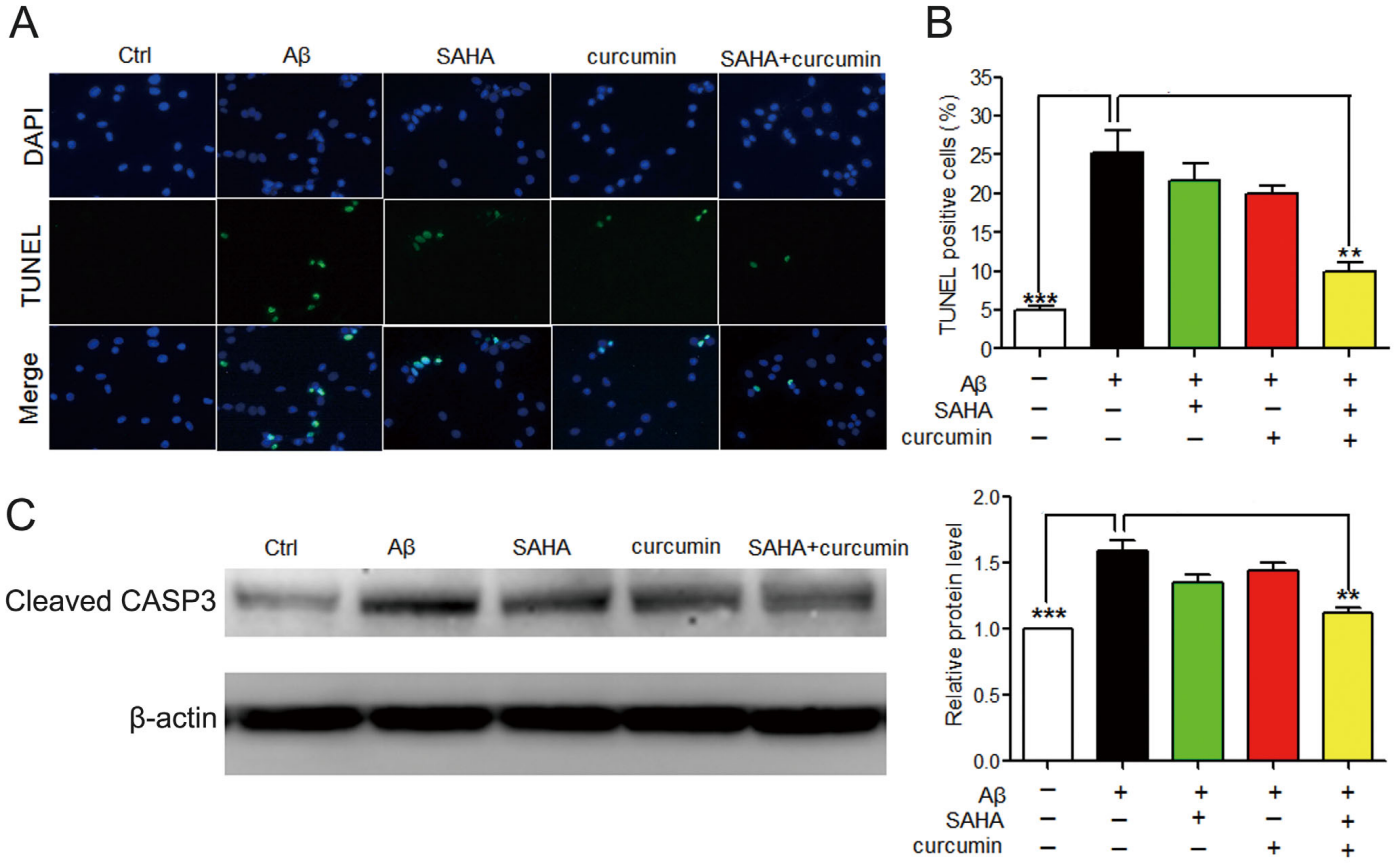
Effect of SAHA and curcumin on cell apoptosis of PC12 cells. A. Apoptotic cells were detected by TUNEL assay. Cells were stained with TUNEL positive nuclei (green) and nuclei of PC12 cells (blue). B. The percentage of TUNEL positive cells was determined (n = 5). ***, *p*<0.001 vs Ctrl; **, *p*<0.01 vs Aβ_25–35_. Ctrl: the control group. C. Western blot of cleaved caspase 3 (n = 3). ***, *p*<0.001 vs Ctrl; **, *p*<0.01 vs Aβ_25–35_. Ctrl: the control group; CASP3: caspase 3.

**Figure 4:**
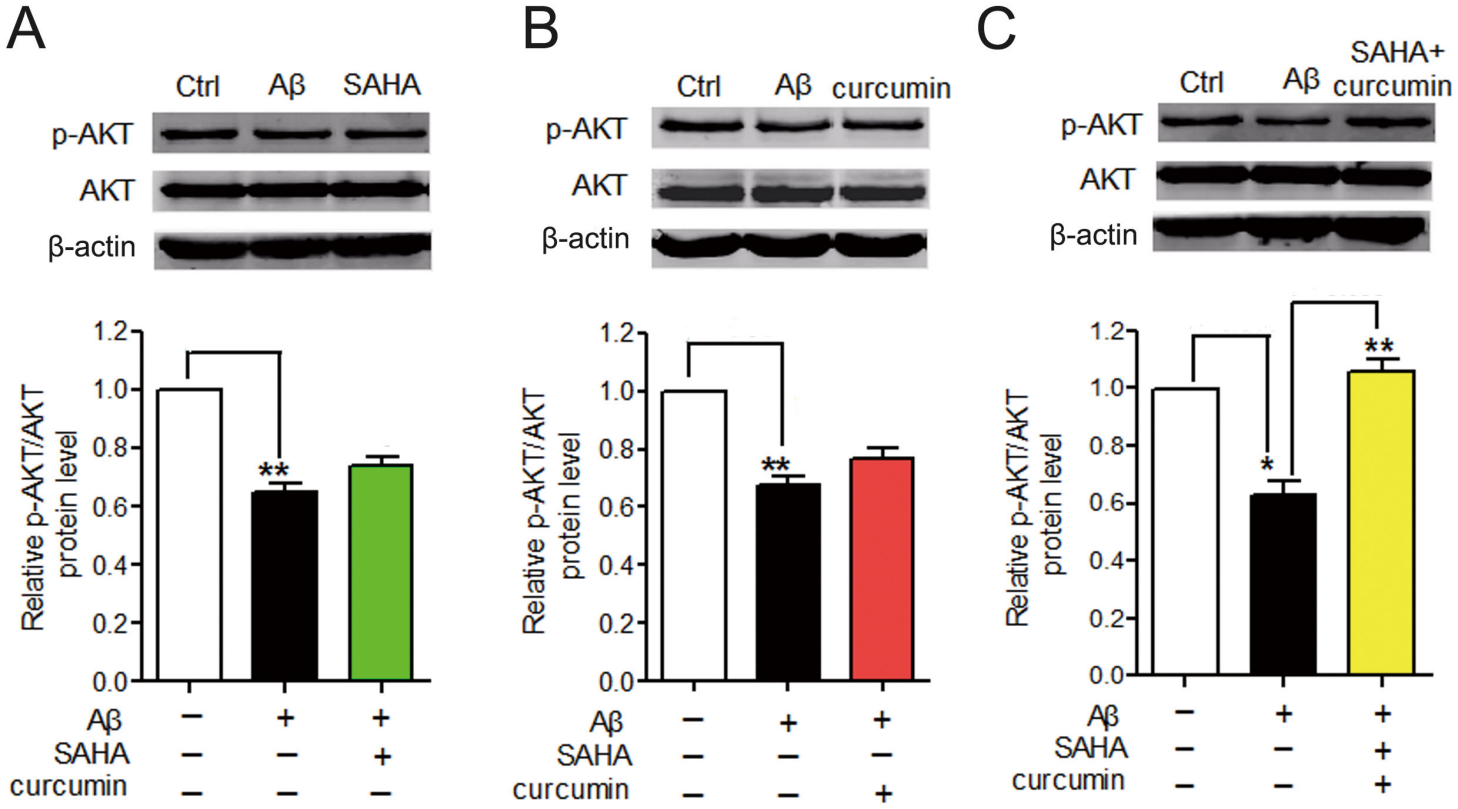
Changes of Akt phosphorylation in PC12 cells after SAHA, curcumin or SAHA+curcumin treatment. A. Representative western blot of the *p*-Akt (Ser 473) protein expression with SAHA treatment (n = 3). **, *p*<0.01 vs Ctrl. B. Representative western blot of the *p*-Akt (Ser 473) protein expression with curcumin treatment (n = 3). **, *p*<0.01 vs Ctrl. C. Representative western blot of the *p*-Akt (Ser 473) protein expression with co-treatment of SAHA and curcumin (n = 3) to the PC12 cells (n = 3). *, *p*<0.05 vs Ctrl; **, *p*<0.01 vs Aβ_25–35_; Ctrl: the control group.

The results of TUNEL staining assay and Western blot analysis of cleaved caspase 3 further validated a synergistic protective effect exists between SAHA and curcumin against Aβ_25–35_-mediated cell apoptosis in PC12 cells (Figure 3). Similarly, we found that co-treatment of SAHA and curcumin, but not SAHA or curcumin alone, provided an obvious anti-apoptotic effect against Aβ_25–35_-induced neuronal injury. The result of the TUNEL staining assay was consistent with that observed in AO/EB staining (Figure 2A). It has been reported that inhibition of Akt activity could induce neuronal apoptosis [24]. Thus, we would expect to see an increase in Akt activity in the presence of SAHA and curcumin. In our study, we validated an apparent improvement of Akt activity and a decrease in apoptosis by the novel SAHA and curcumin combination and that this improvement might be through the synergistic enhancement of *p*-Akt1 activity (Figure 4).

### Both CREBBP and EP300 Maintain Significantly Correlated Expression as AD Evolves

**Figure 5:**
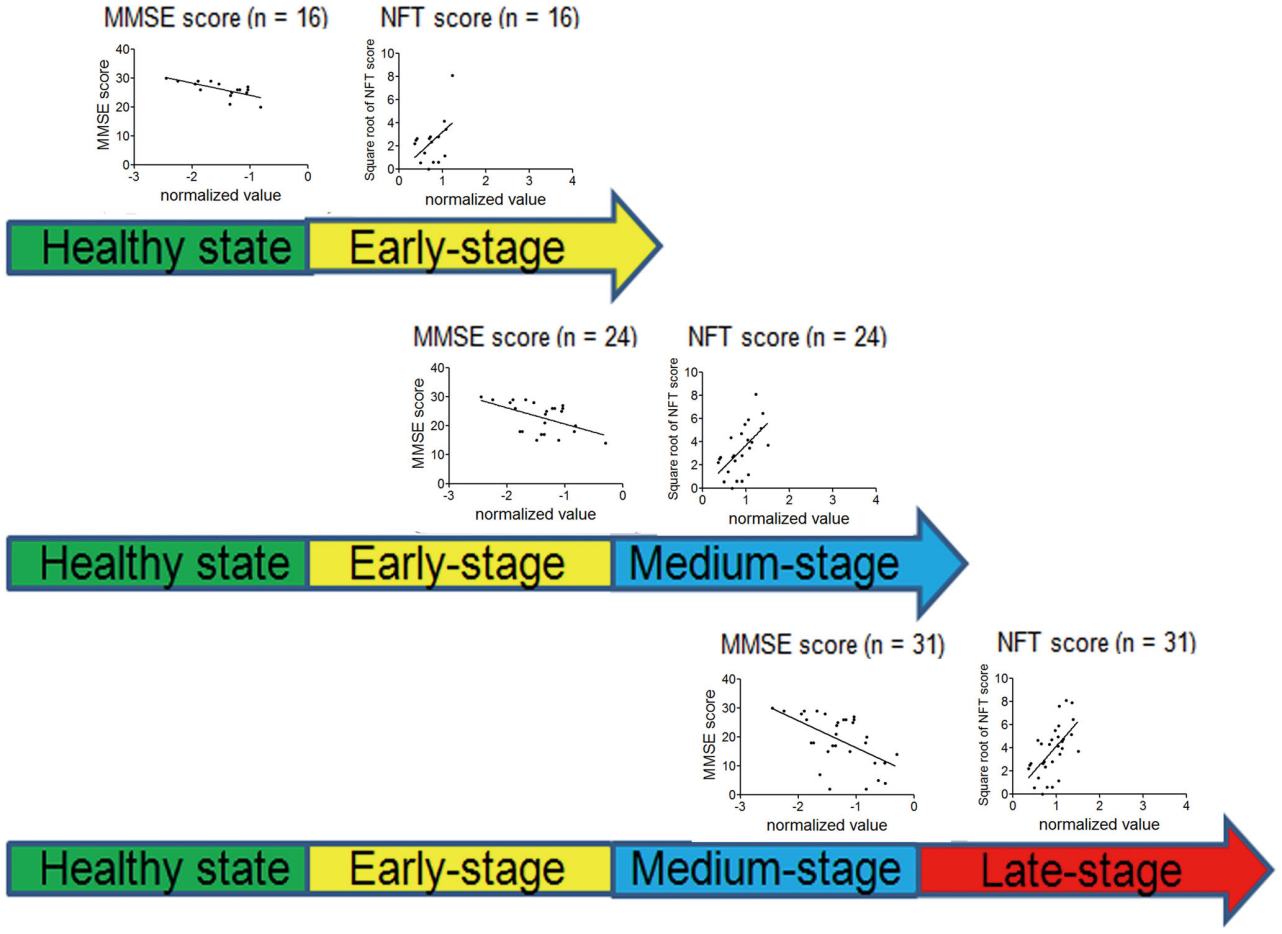
Illustration of gene expression correlation analysis with CREBBP (MMSE score) and EP300 (NFT score) as example. MMSE score:MiniMental Status Examination score; NFT score: neurofibrillary tangle score. Normalized value: the median-normalized gene expression value (log_2_).

Gene expression correlation analysis was performed to obtain the AD gene profiles of different disease stages. The genes CREBBP and EP300 were used as examples for the methodology illustration (Figure 5). CREBBP and EP300 genes have been reported to be associated with AD and their expression significantly correlated with MMSE score and the square root of NFT score, respectively (R^2^>0.30, adjusted *p*-value <0.05). We identified a total of 862 AD-related protein-encoding genes. More AD-related genes were significantly correlated with the square root of the NFT score (n = 329) than with the MMSE score (n = 115) at the early-stage of AD. This finding was consistent with the fact that there is little MMSE decline in patients with early AD [25].

**Figure 6:**
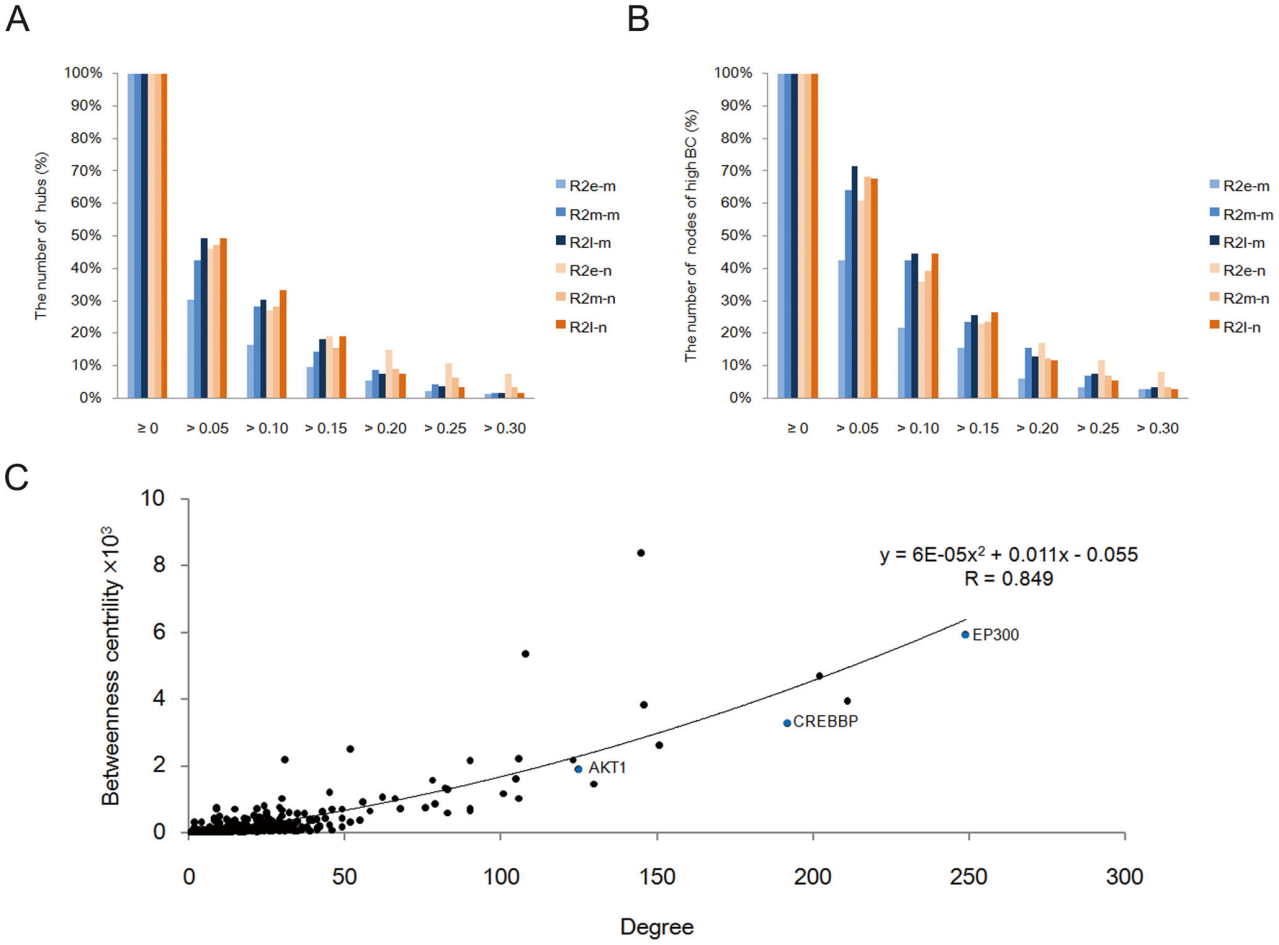
Results of network topology calculation for AD-related genes. A. Statistical result of R^2^ for hub-encoding genes (degree >50) at different thresholds. B. Statistical result of R^2^ for genes encoding proteins of high betweenness centrality (betweenness centrality >5×10^−4^) at different thresholds. C. Strong binomial correlation was found between the topological parameters degree and betweenness centrality (R = 0.849). Three AD-related genes AKT1, CREBBP and EP300 were highlighted as blue nodes. R^2^_e-m_, R^2^_m-m_, R^2^_l-m_: R^2^ with MMSE score at early-, medium-, and late-stage of AD, respectively; R^2^_e-n_, R^2^_m-n_, R^2^_l-n_: R^2^ with square root of NFT score at early-, medium-, and late-stage of AD, respectively.

After calculating the topological parameter degree for all nodes in the hHPIN (6829 nodes and 40478 edges), we found that there were only several hub-encoding genes maintaining significant expression correlation with MMSE score or with the square root of NFT score during the progression of AD (Figure 6A). We focused on hub-encoding genes due to their vital roles in cellular functions [26], [27]. Despite these were a small number of genes, aberrant expression of these hub genes suggested that their greatest responsibility is the initiation and evolution of AD pathology. Furthermore, due to the potential critical roles of proteins of high betweenness centrality in AD [28], we also assessed the AD-related genes that encoded proteins of high betweenness centrality using gene expression correlation (Figure 6B). A strong binomial correlation was found between degree and betweenness centrality (Figure 6C). The MMSE score was responsible for only one gene that encoded hub of high betweenness centrality, EP300 (R^2^_e-m_ = 0.50, R^2^_l-m_ = 0.31), and the NFT score for another three genes including CREBBP (R^2^_m-n_ = 0.34, R^2^_l-n_ = 0.36), EIF6 (R^2^_e-n_ = 0.47, R^2^_m-n_ = 0.32), and NEDD8 (R^2^_e-n_ = 0.42, R^2^_m-n_ = 0.31). CREBBP and EP300 encoded two hub proteins CBP (CREB binding protein) and p300 (E1A binding protein p300), respectively. Our finding implies that the CBP/p300 signaling pathway might play a pivotal role in the progression of AD [13].

### Restoring a Functional Link between Crucial AD-related Processes may Benefit AD

**Figure 7:**
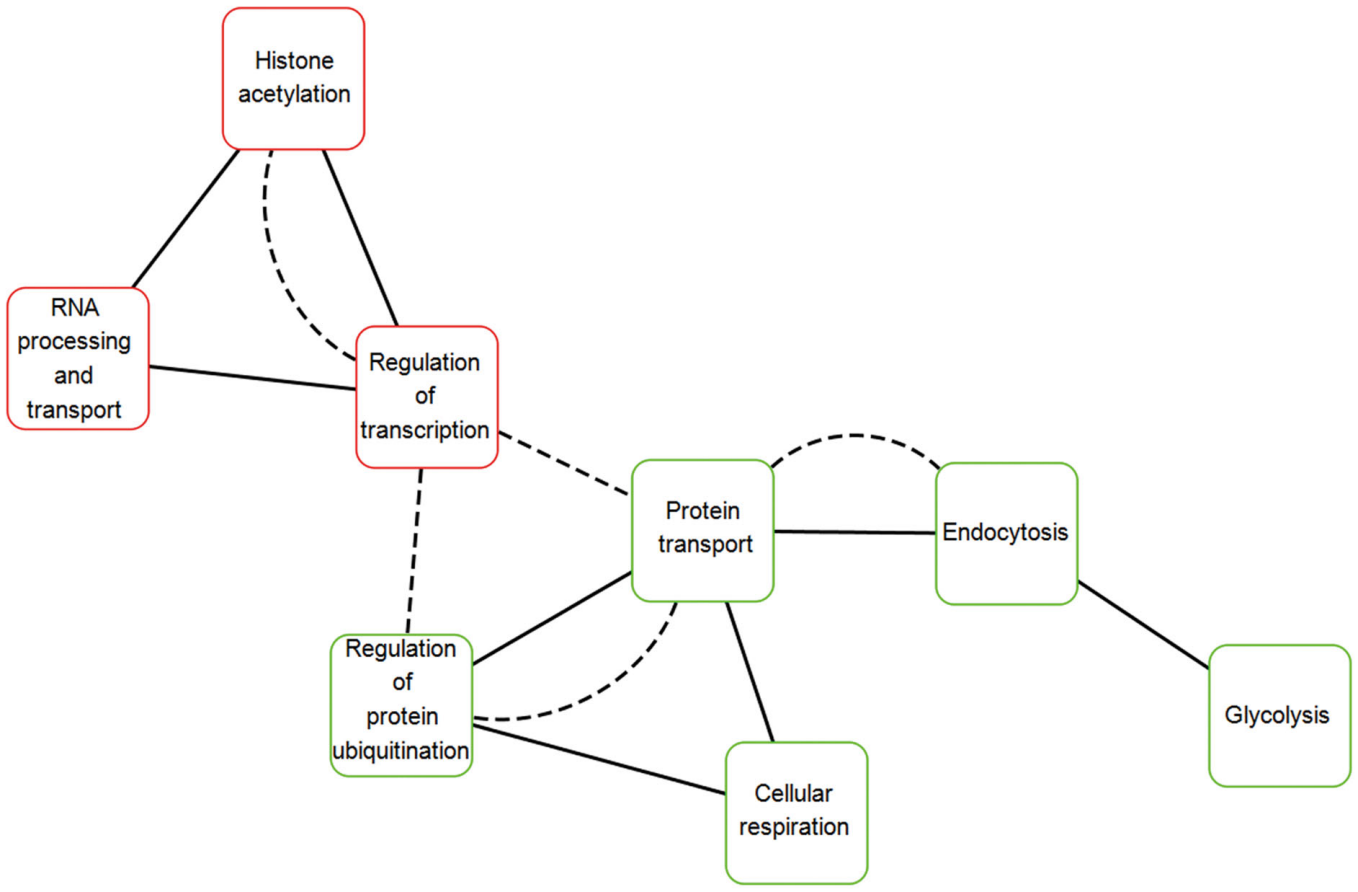
Results of GenePro and expression distance analyses. The AD-related processes of up- and down-regulation were distinguished using red and green border colors. If gene expression distance was less than 0.20, two nodes representing AD-related processes were connected with edge by solid line. If PPIs was more than 20, them were connected with edge by dashed line.

GO analysis revealed that eight processes were significantly over-represented in AD pathology (Table 1). The emergence of regulation of transcription, histone acetylation, and RNA processing and transport suggested the continuous adjustment of cellular transcriptional behaviors; whereas, involvement of glycolysis, cellular respiration, and endocytosis suggested gradual weakening of cellular energy supply and neuronal functions. We also disclosed the functional association between AD-related processes by applying GenePro (Figure 7). Two up-regulated processes, regulation of transcription and histone acetylation, were the most closely interacted [58 protein-protein interactions (PPIs)] among all of the process pairs, followed by the down-regulated process pair, protein transport and regulation of protein ubiquitination (40 PPIs). Strong functional links between the above process pairs suggested that their process genes were functionally complementary to each other. The presence of a high number of PPIs between the up-regulated process of regulation of transcription and the two down-regulated processes, protein transport (33 PPIs) and regulation of protein ubiquitination (22 PPIs), implies there is an impaired functional connection between them during AD.

**Table 1:** Significant over-represented biological processes in AD.

Gene expression	Processes (ES, sum of genes)	Early (%^a^)	Medium (%)	Late (%)
Up-regulated	Regulation of transcription (3.38, 99)	40 (27.0%)	54 (39.7%)	48 (37.5%)
	Histone acetylation (2.28, 19)	7 (4.7%)	9 (6.6%)	10 (7.8%)
	RNA processing and transport (2.20, 30)	11 (7.4%)	16 (11.8%)	11 (8.6%)
Down-regulated	Protein transport (4.04, 69)	32 (21.6%)	19 (14.0%)	24 (18.8%)
	Glycolysis (3.05, 13)	9 (6.1%)	3 (2.2%)	3 (2.3%)
	Cellular respiration (2.20, 37)	14 (9.5%)	12 (8.8%)	13 (10.2%)
	Endocytosis (2.12, 26)	16 (10.8%)	7 (5.1%)	9 (7.0%)
	Regulation of protein ubiquitination (2.02, 38)	19 (12.8%)	16 (11.8%)	10 (7.8%)

**Significant over-represented biological processes in AD.** ES: Enrichment Score; a: the relative percentage proportion of the number of genes in each process was calculated at early-, medium-, late-stage of AD, respectively.

**Figure 8:**
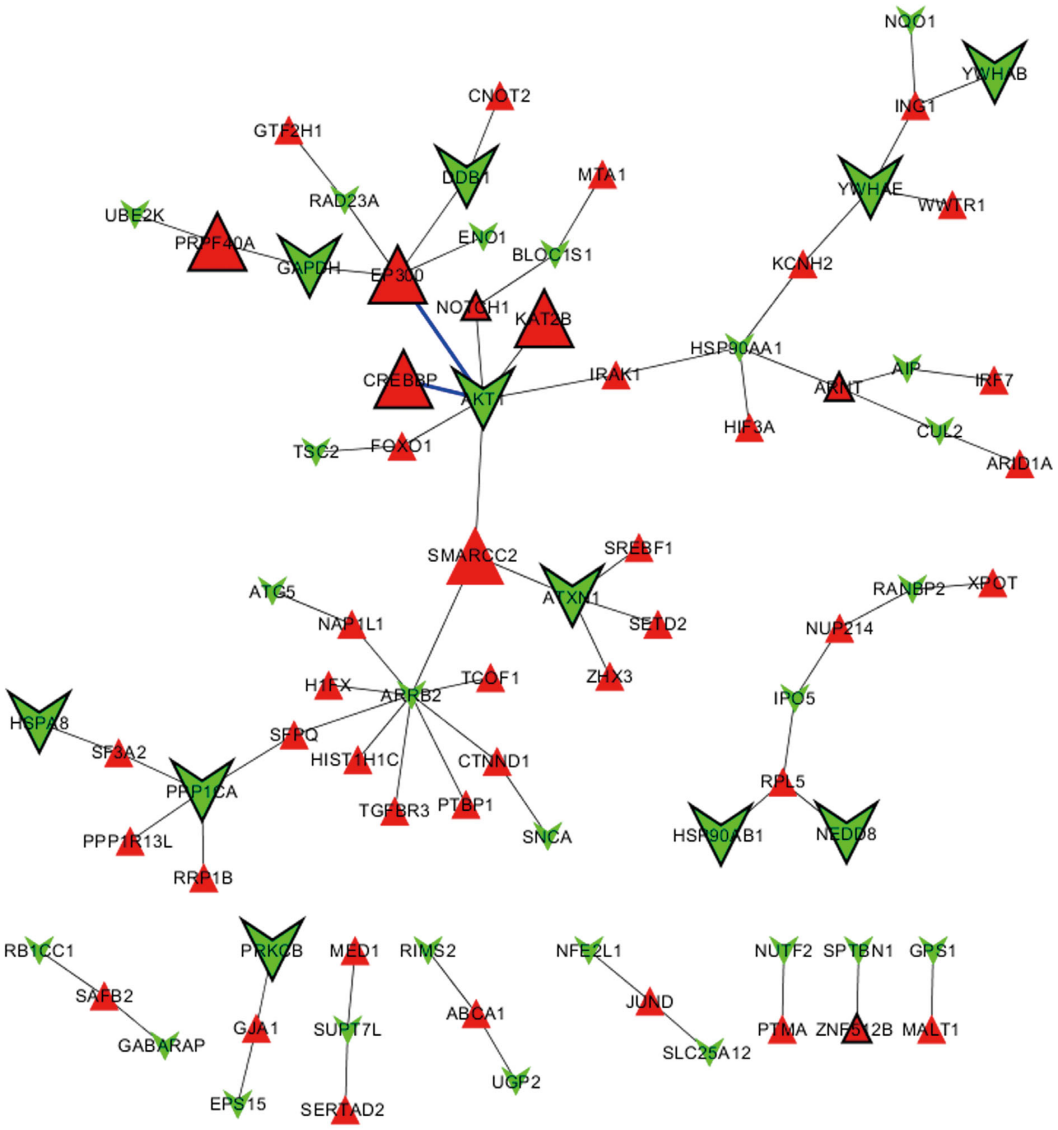
Network visualization of interactions between proteins encoded by up-regulated AD process genes (red triangle nodes) and those encoded by down-regulated AD process genes (light green rectangle nodes). Big nodes represent the hBPIN hub proteins that encoded by AD-related genes (degree >50). The black node border represents that the betweenness centrality of the protein is high (betweenness centrality >5×10^−4^). Functional interactions between protein Akt and the hub proteins CBP and p300 were highlighted as thick blue edges, which were encoded by AKT1, CREBBP and EP300, respectively.

**Figure 9:**
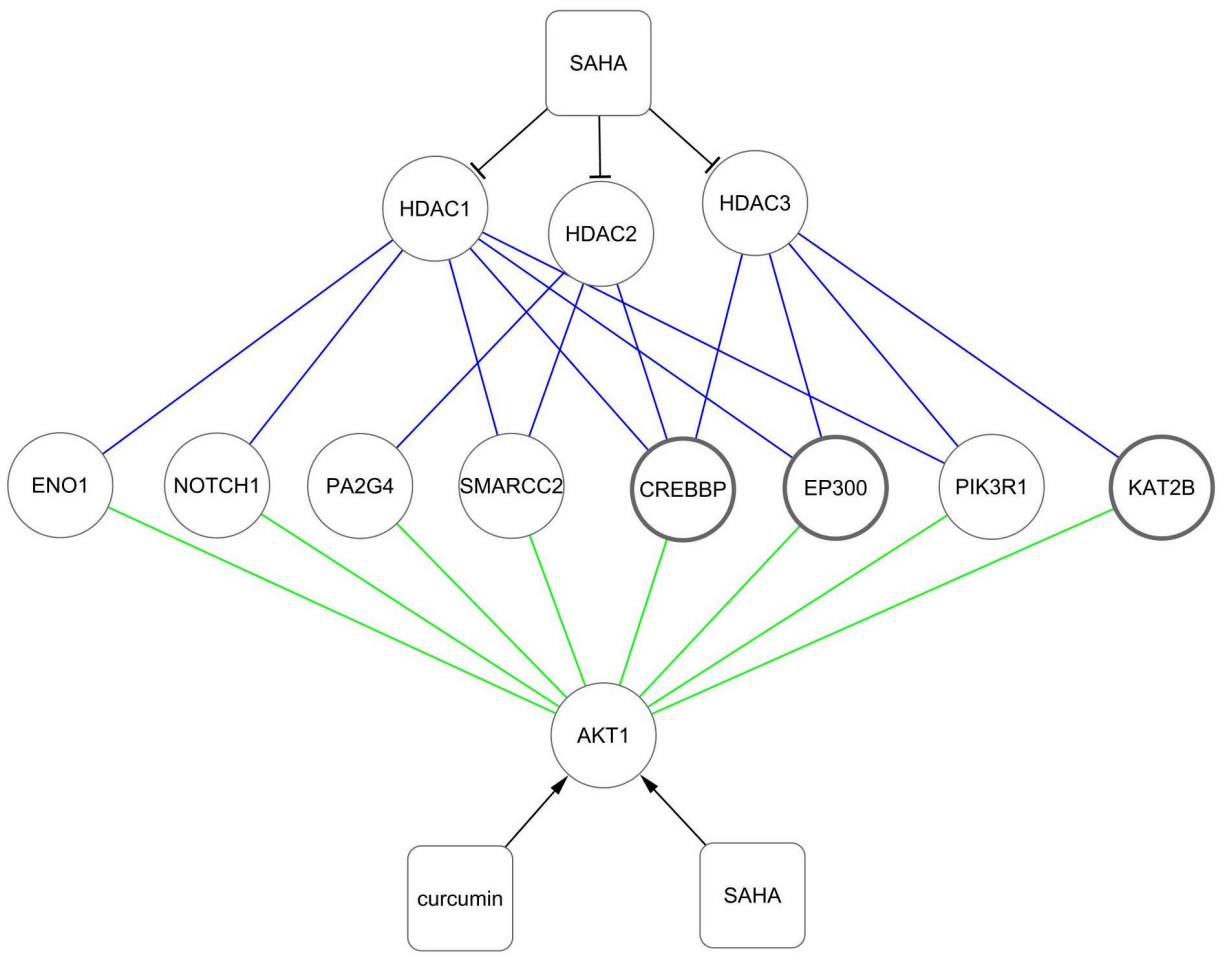
Network visualization of potential mechanism underlying synergistic cytoprotection by SAHA and curcumin. SAHA is a non-selective inhibitor of HDACs and along with curcumin synergistically activated Akt. Eight proteins encoded by AD-related genes were found to be simultaneously associated with HDACs-encoding proteins (blue edges) and Akt which is encoded by AKT1 (green edges) in hBPIN. Nodes with dark lines represent hubs of high betweenness centrality in hBPIN.

Aberrant gene expression reflected continuous alteration of processes as AD developed. Gene expression distance analysis measured whether two AD-related processes would likely be co-expressed during AD. Results indicated that gene expression orientation really produced an obvious impact on gene co-expression (Figure 7). More importantly, it strongly suggested the remarkable imbalance of the up- and down-regulated AD-related processes. Uncoordinated gene expression may more seriously affect the processes that are closely functionally, especially when their links are dependent on a large number of PPIs. Uncoordinated gene expression indicates persistent weakness in the functional connection between the mentioned processes in AD pathology. Thus, we proposed that functional restoration of the link between the up- and down-regulated processes might greatly benefit AD. Furthermore, by visualizing PPIs, we found that the hub protein, Akt was located at the center of the network and functionally interacted with CBP and p300 (Figure 8). The corresponding genes CREBBP and EP300 were involved in the two up-regulated processes, regulation of transcription and histone acetylation. The AD-related gene AKT1 encodes protein Akt, which was involved in all of the down-regulated processes except endocytosis. Network analysis further revealed that due to the synergistic activation of Akt activity with curcumin (Figure 4) and inhibition of HDACs by SAHA [12] joint application of SAHA and curcumin may restore the damaged functional link between crucial disease-related processes in AD. Encoding hubs of high betweenness centrality highlighted the important role of AD-related genes CREBBP and EP300 (Figure 6b, 9) in the synergistic protection against Aβ_25–35_-mediated cell apoptosis.

## Discussion

With aging of the population, neurological diseases such as AD might make our health care system and even whole society become overburdened in the near future [4]. Medicines for the treatment of AD in today's clinics are mainly acetylcholinesterase inhibitors such as rivastigmine and the low-affinity NMDA receptor antagonists such as memantine. Current medications for symptomatic relief and the lack of effective control for disease progression have made AD treatment strategy particularly embarrassing, especially when facing the serious epidemiology of AD [29]. Undoubtedly, the development and clinical accessibility of effective AD pathology-reversing drugs is of urgent need to the diagnosed and at risk AD patients.

Currently, among the potential pathological mechanisms and intervention strategies that have been proposed as promising AD treatments, tuning acetylation levels with HDAC inhibitors or HAT activators is highlighted and widely recognized by researchers [10], [14]. However, recent clinical failures of Aβ-directed therapeutics clearly remind us that there are more than one molecular pathway simultaneously implicated in the complex pathogenesis and development of AD [1]. Intervention of a single pathological pathway may be not enough to effectively reverse or slow down the disease’s progress due to the interaction of several biological systems such as network robustness, redundancy, crosstalk, et al. [30]. Thus, the simultaneous intervention of multiple pathological pathways is a more preferable therapeutic strategy especially for AD [31], [32].

In this study, we demonstrated for the first time a potent synergistic action of the anti-tumor HDAC inhibitor, SAHA, with the natural flavonoid, curcumin, on Aβ_25–35_-induced neuronal injury in PC12 cells. No significant neuroprotection against Aβ-induced decrease of cell viability and apoptosis was found by using 1 µM of SAHA or 5 µM of curcumin alone. However, co-administration of SAHA and curcumin improved neuronal viability and successfully restored the structural integrity of the neuronal cells damaged by Aβ treatment. Inhibition of HADC activity and fine-tuning histone acetylation levels had been previously shown to be the mechanisms underlying the role of SAHA in AD [33]. Comparably, some molecular mechanisms different from SAHAs are involved in the neuroprotective effect of curcumin [34]. Thus, we proposed that the potent synergy between SAHA and curcumin might not be through the same molecular pathway but through the intrinsic linkage between two independent mechanisms [15], [35].

Recently, network biology, an emerging research branch of systems biology, has been applied to re-define the neurological diseases including AD from a biological network perspective [36]–[38]. Here, a comprehensive network analysis of global gene expression changes in AD [7] was performed in order to reveal the causal links between pathological processes that promote AD evolution. Among the identified AD-related genes that encoded hubs in the hHPIN, the CBP/p300 signaling genes, CREBBP and EP300, were both highlighted due to the stable correlation of their expression with MMSE score and the square root of NFT score, respectively. The influence of hub proteins over the PPIs in the hHPIN strongly suggests that CBP/p300 signaling might not be the only histone acetyltransferase and deacetylase balance that is crucial for progressive neurodegeneration [14], but it may also be implicated in the maintenance of normal neuronal functions [39].

Consistent with the previous finding [7], our result further confirmed that significant alterations in expression of transcriptional genes was associated with the onset of AD instead of those genes that directly participated in the maintenance of cellular energy supply and neuronal functions. This result supports the fact that the available pharmaceutical therapies such as an acetylcholinesterase inhibitor and glutamate receptor antagonist can only ameliorate cognition of AD patients but fail to reverse the AD pathology [40]; while, the HDAC inhibitor, which affects transcriptional activity, is able to enhance neuronal survival and slow or prevent progressive neurodegeneration [33] and it has beneficial effects on learning and memory [10]. We also investigated the gene co-expression of all of the pathological processes in AD and found that pathological processes would likely co-alter as AD evolved, which partly hints that the two participating processes need to be co-expressed to jointly fulfill some cellular functions. For example, all of the up-regulated pathological processes co-altered in gene expression due to the demand of functional coordination. Although the down-regulated pathological processes were relatively isolated for loss of direct PPIs as GenePro analysis suggested, gene co-expression might still reflect functional coordination between each other.

Uncoordinated gene co-expression of the important AD-related processes regulation of transcription, protein transport and regulation of protein ubiquitination during AD progress suggests pathological weakening of the functional link between them [41]. Thus, restoration of the functional link between the above pathological processes is very important for the reversal of AD pathology. Critical PPIs were successfully revealed by establishing a network visualization of functional links between processes. Enhancing the activity of the CBP/p300 signaling pathway or Akt should be equally reasonable for firming the association between processes.

By targeting the CBP/p300 signaling pathway with small molecule HADC inhibitors, one study showed that neuronal survival could be enhanced and the progressive neurodegeneration slowed or prevented and memory impairments could be reversed, which is associated with the early-stage of AD [11]. However, our experiment demonstrated that with the increase concentration of SAHA, neuron cells might be subject to significant cytotoxicity. This finding was consistent with the reasonable worries about the specificity of this strategy [13]. However, we found that curcumin could markedly improve the therapeutic specificity of SAHA via synergistically relieving the Aβ_25–35_-induced neuronal injury by targeting Akt [34]. This is consistent with network analysis results showing that the damaged functional link between Akt and CBP/p300 pathways may play a causative role in the evolution of AD. Our study proves that by representing the disease as a biological process network can provide novel insights into the mechanism underlying AD and facilitate identification of potential therapeutic targets.

Taken together, our study suggests that the proposed synergistic combination of SAHA and curcumin should be fully considered for AD treatment in the future. More importantly, compared to a monotherapy, a drug combination with synergistic action mechanisms would bring more advantages, such as being less prone to drug resistance, more forcefully confrontational against biological network robustness, smaller drug dose with less side effects, and improved disease-relevant therapeutic effect and selectivity, especially when complex pathological mechanisms are involved in the progression of the disease [15], [30], [42]. Furthermore, instead of a huge investment and the lengthy process in drug development, rational utilization of the current drugs clinically available may be a more economical strategy; thus, a viable therapeutic approach could be proposed within a relatively short period of time. Further functional experiments in vivo are definitely needed, especially, regarding the therapeutic effect and safety between the novel combination, SAHA and curcumin, for the treatment of AD.

In summary, the HADC inhibitor, SAHA, represents a hopeful therapeutic agent for AD in the future. However, some technical issues remain to be resolved; prominently, its poor therapeutic selectivity due to its broad-spectrum inhibition of HDACs. Because different molecular mechanisms are simultaneously involved in the pathological complexity of AD, this leads to the possibility and rationality of a potential drug combination therapy by exerting a synergistic pharmacological effect. In this manner, on one hand, the drug dose might be substantially reduced with less drug side effects; on the other hand, the higher therapeutic selectivity of medicine jointly used. Our in vitro experiment confirmed an outstanding improvement of Aβ-induced neuronal injury by synergistic action of SAHA and the natural flavonoid, curcumin. This finding strongly implies the feasibility of the non-selective HADC inhibitor, SAHA, for clinical AD treatment in the future, specifically as a novel means of SAHA-centered combination therapy with other drugs clinically available, such as nutritional supplements with curcumin as the main ingredient.
